# Colonizing the High Arctic: Mitochondrial DNA Reveals Common Origin of Eurasian Archipelagic Reindeer (*Rangifer tarandus*)

**DOI:** 10.1371/journal.pone.0165237

**Published:** 2016-11-23

**Authors:** Kjersti S. Kvie, Jan Heggenes, David G. Anderson, Marina V. Kholodova, Taras Sipko, Ivan Mizin, Knut H. Røed

**Affiliations:** 1 Department of Environmental Studies, University College of Southeast Norway, Bø in Telemark, Norway; 2 Department of Basic Sciences and Aquatic Medicine, Norwegian University of Life Sciences, Oslo, Norway; 3 Department of Anthropology, University of Aberdeen, Aberdeen, Scotland; 4 A.N. Severtsov Institute of Ecology and Evolution RAS, Moscow, Russia; 5 Russian Arctic National Park, Arkhangelsk, Russia; CSIRO, AUSTRALIA

## Abstract

In light of current debates on global climate change it has become important to know more on how large, roaming species have responded to environmental change in the past. Using the highly variable mitochondrial control region, we revisit theories of *Rangifer* colonization and propose that the High Arctic archipelagos of Svalbard, Franz Josef Land, and Novaia Zemlia were colonized by reindeer from the Eurasian mainland after the last glacial maximum. Comparing mtDNA control region sequences from the three Arctic archipelagos showed a strong genetic connection between the populations, supporting a common origin in the past. A genetic connection between the three archipelagos and two Russian mainland populations was also found, suggesting colonization of the Eurasian high Arctic archipelagos from the Eurasian mainland. The age of the Franz Josef Land material (>2000 years before present) implies that Arctic indigenous reindeer colonized the Eurasian Arctic archipelagos through natural dispersal, before humans approached this region.

## Introduction

Climatic oscillations over the Quaternary (2.4 million years ago–present) have had a major impact on the geographic distribution and genetic structure of species through population extinctions and range shifts [1]. The demographic impact of range shifts alters the genetic structure of populations by the elimination of populations and lineages, reduction in genetic variation due to bottlenecks and founder events, as well as the spread of mutations by selection and population expansion [2]. How individual populations respond to such changes varies with their environmental tolerance, their ability to adapt [3] and their capacity to disperse to accommodate the rate of environmental change [4]. Arctic landscapes pose a particular challenge for terrestrial mammals due to the vastness and the way that intermittent ice-cover, oceans, and topography fragment the landscape. Furthermore, Arctic species are considered particularly vulnerable to climate changes, as even small changes may result in immediate and long-lasting effects [5].

Reindeer and caribou (*Rangifer tarandus*) is a keystone species in the circumpolar North, not only ecologically through the way they impact upon the plant cover [6], but also as a source of subsistence to local residents and more recently as a focus for defining protected areas [7]. Fossil evidence shows that during the Pleistocene, *Rangifer* was distributed south of the ice sheet in both Eurasia and in North America, and in the Beringia refugium encompassing the Bering land bridge, Alaska, as well as large parts of Siberia [8]. *Rangifer* exhibit distinct morphological adaptations to different environments, and populations have been subdivided into various ecotypes according to their life-history strategies and ecological conditions such as the woodland or boreal forest or sedentary form, the barren-ground or tundra or migratory form, the mountain form, and the Arctic form [8]. The Arctic form, thought to be better adapted to cold, open environments, is usually recognized by its small body size, with short rostrum and legs, as well as a thicker, paler winter pelage [9]. The morphologically-based Arctic type is made up of several populations: Svalbard reindeer (*R*.*t platyrhynchus*), distributed on the Svalbard archipelago, the North American Peary caribou (*R*.*t pearyi*) primarily distributed on the Canadian Arctic archipelagos, and the recently extinct *R*.*t eogroenlandicus*, formerly distributed on Eastern parts of Greenland [10]. In the Arctic, also less morphologically distinct reindeer exists as those on Novaia Zemlia in northern Russia. Russian taxonomists classify the reindeer inhabiting the Novaia Zemlia archipelago as members of the tundra type [11–15] and have since 2001 been registered by the Russian Federation as a geographically isolated subspecies (*R*.*t*. *pearsoni*) with a view to restoration [16, 17].

There has been a wide-ranging debate on the colonization routes and dispersal of Arctic reindeer inhabiting the islands in the western Eurasian and North American Arctic [8, 9, 18, 19]. Mitochondrial DNA (mtDNA) has shown to be a highly useful marker to describe past extinction and range expansions on near-present evolutionary time scales [20]. Based on contemporary- and ancient mtDNA, different refugia and colonization routes have been suggested for a range of roaming terrestrial arctic species like the collared lemming (*Dicrostonyx groenlandicus*) [21], reindeer (*Rangifer tarandus*) [19], the Arctic fox (*Alopex lagopus*) [22] and the wolverine (*Gulo gulo*) [23]. Svalbard reindeer, Peary caribou and *R*.*t eogroenlandicus* have been shown to comprise of mtDNA haplotypes signalling a common origin in an ancient Beringian and/or Eurasian pre-glacial population [19]. Recent genetic studies of Novaia Zemlia reindeer have demonstrated the same [14]. The fact that Peary caribou and *R*.*t eogroenlandicus* shared certain mtDNA haplotypes, morphological similarities, as well as have been observed to migrate from Ellesmere Island to North Greenland, provides convincing evidence for a North American colonization route for these two subspecies [9]. However, the colonization route of the existing archipelagic Svalbard and Novaia Zemlia reindeer populations has remained an open question.

Svalbard reindeer are characterized by low genetic variability indicating isolation, possible bottlenecks, and subsequent genetic drift as important population processes [24, 25]. The Svalbard population is characterized by three control region (CR) haplotypes which were previously thought to be unique to Svalbard [26]. However, the most common of these was also found in northern Québec, supporting the idea that these reindeer might have colonized Svalbard from North America [19]. This idea found support in the previously reported similarities in transferrin polymorphism between Svalbard reindeer and Peary caribou and with both having some similarities with the American woodland caribou [27, 28]. A recent genetic survey of wild reindeer populations on Novaia Zemlia have identified nine distinct CR haplotypes, with six thought to be unique to the archipelago [29] and the others showing links both Westward and Eastward. The population is further characterized to show low levels of variation compared to mainland populations [29]. Grineve͡tskiĭ [30] in 1883 first recorded two different types of wild reindeer distributed on the Novaia Zemlia archipelago–one on the South Island and one on the North Island–and reported that local hunters identified a morphological similarity between the latter and animals living on Svalbard. The existence of two separate “races” on the archipelago was repeated by Sokolov [31]. These earlier observations spoke to a common colonization route for all High Arctic reindeer from Eurasia.

The idea that the wild reindeer on some of the Arctic archipelagos might have been genetically linked to the Eurasian mainland was first raised by Gravlund et al. [9] who found a CR haplotype common on Svalbard in a sample from the wild reindeer population on western part of the Taimyr peninsula. The Taimyr population is considered to be the largest wild reindeer population in Eurasia, covering the northern parts of central and partly western Siberia [32]. An early published observation of a possible contemporary migration of a single reindeer bull from Novaia Zemlia to Svalbard over the winter ice presented a possible route. Nøis [33] speculated that Svalbard may have been colonized by reindeer from the Novaia Zemlia archipelago, situated 770 km south east of Svalbard, using the Franz Josef Land group of islands as a stepping stone [34]. The distance from the Franz Josef Land archipelago to Svalbard is approximately 400 km. Although there are no reindeer surviving on Franz Josef Land today, archaic bones and antlers are widely distributed [9, 35]. Previous radiocarbon dating of reindeer antlers sampled on the archipelago indicates that reindeer occupied Franz Josef Land as early as ~6000 years before present (YBP) [36].

A third account of the colonization of some of the High Arctic islands focusses on human-instigated translocations. Resolving the question of possible human induced translocation is important for the issue of introgression from domestic herds, a key parameter for classifying protection status of populations. There is a relatively broad Russian language literature documenting the human interest in wild reindeer, and the movement of reindeer herds between islands in the Eastern Barents Sea in historic times [16, 37, 38]. These sources point to the hunting of wild reindeer by Pomor or Viking coastal dwellers in Novaia Zemlia archipelago from the 12^th^ century onwards [39]. There is one record of domestic reindeer being translocated to the islands by an academic expedition in 1896 [40], although local Nenets families living on the South Island of Novaia Zemlia in the 19^th^ century were recorded as living without domestic reindeer [38]. There are well-documented attempts of the early Soviet authorities to translocate up to 604 head of domestic reindeer from Kolguev Island between 1928 and 1933 [41]. There are also scattered references to wild reindeer migrating over the ice from the Siberian mainland to Novaia Zemlia [16, 37, 38]. Finally, translocation of domestic reindeer from the Norwegian mainland to Svalbard, during expeditions taking place in 1872 and 1913, are also documented [42]. However, there are no domestic reindeer on Novaia Zemlia or on Svalbard today.

Here, we use the highly variable CR to compare sequence data from contemporary reindeer populations on Svalbard and Novaia Zemlia, with ancient samples from the now extinct population on the Franz Josef Land archipelago. Despite the work done comparing Svalbard reindeer to the mainland *Rangifer* populations in both North America and Eurasia, there has been a conspicuous lack of studies comparing the reindeer populations on each of these three neighbouring archipelagos to each other–the clearest approach to discussing possible common colonization routes. A genetic link between Svalbard, Novaia Zemlia and Franz Josef Land would tell us if these archipelagic populations have a common origin and also help to answer the question of whether or not the current distribution of the Arctic type is caused by natural dispersal or recent human induced dispersal of domestic reindeer. We also included sequence data from reindeer on the nearest population of domestic reindeer situated on Kolguev Island to test for introgression from domestic reindeer. Two wild mainland- and one wild island population from Russia were included to help answer the question of a possible biographical link between the Arctic archipelagos and the Eurasian mainland.

## Material and Methods

### Study populations

**Fig 1:**
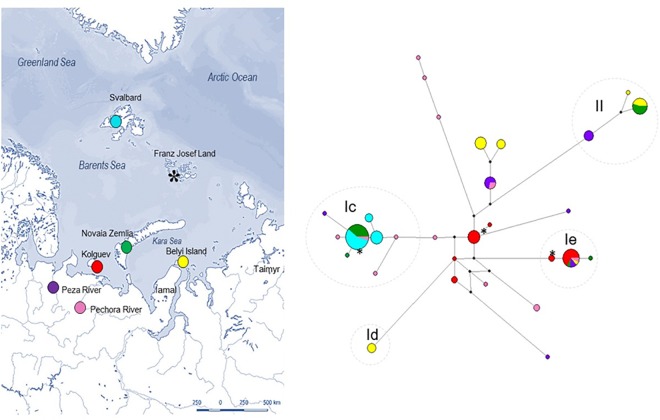
Sampling locations and phylogenetic network showing genealogical relationships in the CR between reindeer populations. Map of Northern Eurasia, with focus on the Eurasian Arctic archipelagos, showing the geographic origin of the samples (a) and a MJ network of the 122 CR sequences (400 bp) (b). Five previously described haplotype clusters (**Ic**, **Id**, **Ie**, and **II**) [26, 69] are identified. The MJ network show haplotype sharing between Svalbard (turquoise), Novaia Zemlia (green) and Pechora River (pink) within sub-cluster **Ic**. Including the Franz Josef Land samples (asterisk) show that 13 of the 15 ancient samples sequenced were identical to the most common haplotype found on Svalbard and on Novaia Zemlia. We also found one individual with a haplotype belonging to sub-cluster **Ie**, and one haplotype that is unique for Franz Josef Land. The map (a) is printed here for the first time under a CC BY license, with permission of the cartographer Allessandro Pasquini.

Blood-, muscle- and archaic antler samples were obtained from arctic populations on Svalbard (n = 3, in addition to 24 sequences downloaded from GenBank), Novaia Zemlia (n = 20) and Franz Josef Land (n = 15.) The Svalbard population was sampled at Nordenskiöld Land on Spitsbergen and on Nordaustlandet. Wild reindeer samples from Novaia Zemlia were collected on the South Island (Fig 1A, S2 Table). Archaic antlers were collected on Hooker- and Hays islands on the Franz Josef Land group. Four of these samples were ^14^C dated (S1 Table). Skin- and velvet samples were collected from wild reindeer on Belyi Island (n = 22), and from domestic reindeer from Kolguev Island (n = 24). Belyi Island lies 430 km to the south and east of Novaia Zemlia and directly North of the Iamal peninsula and has a distinct population of wild reindeer [43, 44]. Kolguev Island lies 254 km Southwest of Novaia Zemlia and has supported Nenets domestic reindeer breeders [45] as well as a history of provisioning Novaia Zemlia with domestic stock in the 19^th^ and early 20^th^ century. Sequences from two continental wild reindeer populations, the Peza River Basin, Peza district, Arkhangelsk oblast’ (n = 6) and the headwaters of the Pechora River in the Pechro-Ilychskii Nature Reserve, Komi Republic (n = 10), were downloaded from GenBank and included in the analyses in order to test for possible gene flow between the mainland and the archipelagos (S2 Table). Additional sequences from these populations were provided by the investigators (n = 13) [46, 47] (S2 Table). The Peza River population is classified as a forest reindeer ecotype and the Pechora River population as a forest-mountain reindeer ecotype [47]. To our knowledge, these populations have not previously been compared with Eurasian arctic archipelagic populations.

### Ethics statement

Blood and muscle samples from Svalbard were collected as part of the Man and the Biosphere (MAB) project which started in 1978. The Svalbard MAB field project was coordinated by the Norwegian Polar Institute (Norway's central governmental institution for scientific research, mapping and environmental monitoring in the Arctic and the Antarctic). Hunting permits of reindeer within the Svalbard MAB field project was approved by the Governor of Svalbard. Sampling of muscle-, skin- and velvet samples from Novaia Zemlia, Belyi Island and Kolguev required no specific permits and was done under an ethics review for the ERC Arctic Domus and performed under the ERC Arctic Domus ethics annex.

Muscle and skin samples of wild reindeer from Novaia Zemlia and Belyi Island were collected from dead animals via subsistence hunting. The sampling was conducted by the authorized managers of these populations, according to the regulations stated by the Ministry of Nature Protection of the Russian Federation. Velvet samples from domestic reindeer on Kolguev Island was collected from dead animals during industrial slaughter. No animals were sacrificed for this study and the field work did not involve endangered or protected species.

### DNA analyses of contemporary samples

Tissue samples were stored in ethanol (≥ 80%) or kept frozen until analysed, blood samples were stored in EDTA. DNA extraction of muscle-, velvet- and skin samples was performed using DNeasy Blood & Tissue Kit (Qiagen) following the manufactures protocol. DNA extraction from EDTA blood was carried out using DNeasy Blood & Tissue Kit (Qiagen) or by using a boiling method for DNA extraction (SI).

A 503 base pair (bp) long fragment from the mitochondrial control region was amplified using the forward primer RtCRF (5`-AAT AGC CCC ACT ATG AGC ACCC-3`) [19] and the reverse primer RtCR-528 (5`-TAG GTG AGA TGG CCC TGA AGA AA-3`) [48]. Amplification was performed using the following program: 95^°^C for 2 min, 95^°^C for 30 sec, 55^°^C for 30 sec and 72 ^o^C for 1 min (step 2–4 cycled 30 times) and finally 72 ^o^C for 10 min. PCR reactions were performed in 20 μl total volume using 1–2 μl DNA template, and with the following final concentrations; 1X buffer, 1.5 mM MgCl_2_, 0.8 mM dNTPs, 5 pmol of each primer, 0.5 μg/μl Bovine Serum Albumin (BSA), 0.5 U/μl AmpliTaq DNA polymerase (Applied Biosystems), and dH_2_O to make up the remaining volume.

The samples were cleaned for unincorporated primers and nucleotides using Illustra ExoProStar (GE Healthcare) diluted 10 times. Cycle sequencing was performed in a 10 μl reaction volume, using BigDye v3.1 sequencing kit (Applied Biosystems) following manufacturer`s recommendations. Purification was carried out using standard EDTA/EtOH precipitation. Capillary electrophoresis and data analysis were performed with an ABI 3130xL- or 3500xL instrument (Applied Biosystems). All sequences were sequenced in both directions and the consensus sequences were aligned by ClustalW [49] and edited in MEGA v5.2 [50]. The sequence alignment was trimmed to 400 bp to be aligned with sequences downloaded from GenBank.

### DNA analyses of ancient samples

DNA was extracted from antler powder using DNeasy Blood & Tissue kit (Qiagen) following Bjørnstad and Røed [48]. Standard precautions for working with ancient samples were undertaken [51, 52]. All equipment and working surfaces were cleaned using sodium hypochlorite, ethanol or UV-light. Samples were mechanically cleaned and the outer surface was removed before drilling out the powder. To test for contamination, blank extraction and PCR controls were used in each PCR reaction and only DNA sequences which could be replicated from at least two independent amplifications of each primer pair were accepted.

From the ancient material a 266 bp fragment of the mtDNA control region was amplified using the primer pair 259F/524R (5’–TGCCCCATGCTTATAAGCAAG–3’/ 5’–GTGAGATGGCCCTGAAGAAA–’3), or by amplifying two overlapping amplicons of respectively 140 bp with primers 259F and 398R (5’- CCTTTCTTGTCAACATGCGTA– 3’) and 178 bp with primers 347 F (5’–TGCCCCATGCTTATAAGCAAG–3’) and 524R. PCR amplification and sequencing were performed as in Bjørnstad and Røed [48]. The sequences were aligned by ClustalW and edited in MEGA v5.2. The sequence alignment was trimmed down to 190 bp.

### ^14^C dating of ancient samples

For a verification of the time horizon, we ^14^C dated 4 antler samples from Franz Josef Land which also amplified successful DNA (S1 Table). All ^14^C dates were calibrated using CALIB 6.1.1 [53] based on the data set IntCal13 [54]. The ^14^C dating of 4 of the 15 ancient samples from Franz Josef Land all revealed an age of more than 2000 years (2468–3835 YBP, S1 Table), suggesting that these samples are from wild, indigenous reindeer.

### Statistical analyses

DNA polymorphism estimates (number of haplotypes, gene diversity and nucleotide diversity) were calculated in DnaSP [55] for the contemporary populations, and for the data set including the ancient antler samples. Genealogical relationships were examined by constructing a Median Joining (MJ) network [56] using Network v4.6 (fluxus-engineering.com). BEAST v1.8.0 [57] was used to construct a Bayesian phylogeny based on the haplotypes identified in the dataset comprising the contemporary populations. We used the HKY G+I substitution model and the substitution rate was set to 58.9%/Myr [58]. The analyses were run for 100 000 000 generations and 10% of the initial samples was removed as burn-in. Convergence was assessed in TRACER [59] and the effective sample size for all parameters were above the general recommendation (ESS> 200). We used Arlequin v.3.5 [60] to test for recent demographic expansion of the sub-cluster dominating on the Arctic archipelagos by calculating the mismatch distributions of pairwise nucleotide differences [61, 62], as implemented in Arlequin and with 10 000 bootstrap replicates. For the same sub-cluster, Arlequin was used to calculate the sum of squared deviations (SSD) to test if the observed distribution deviated significantly from the expected under the population expansion model. The Harpending Raggedness index [63] was calculated to check for demographic changes. A smooth morphology indicates a population expansion, whereas a ragged morphology indicates constant population size [63]. Arlequin was used to calculate Fu`s Fs [64] and Tajima`s D [65] to check for deviations from neutrality. We used DnaSP to estimate a third neutrality test, the Ramos-Onsin’s and Roza’s [66] R_2_ value, which may be more appropriate when dealing with small sample sizes. Haplotype frequencies in each of the seven populations were calculated in Arlequin.

## Results

A total of 137 samples from seven populations were analyzed for the mitochondrial control region, including 122 contemporary- and 15 ancient samples. The 400 bp long alignment, comprising the contemporary populations, varied from low to relatively high levels of genetic diversity with a total of 30 haplotypes, overall haplotype diversity (Hd) = 0.910, and nucleotide diversity (π) = 0.019 (Table 1). The Svalbard population exhibited the lowest level of variation (Hd = 0.570, π = 0.002) showing 3 haplotypes. The Novaia Zemlia population showed an intermediate level of genetic diversity (Hd = 0.632, π = 0.018) and in our study 5 haplotypes were identified. The domestic population from Kolguev Island showed an intermediate level of haplotype diversity and low levels of nucleotide diversity (Hd = 0.728, π = 0.009) and 6 haplotypes were found. The wild population from Belyi Island exhibited relatively high levels of variation (Hd = 0.814, π = 0.018) with 6 haplotypes identified. The two wild mainland populations from the Peza and Pechora river basins also showed high levels of variation (Hd = 0.800, π = 0.018 and Hd = 0.978 π = 0.019, respectively) with 6 haplotypes found in the Peza population and 12 in the Pechora population (Table 1).

**Table 1:** Geographic origin, status (wild/domestic) and polymorphism in the CR in the sampled reindeer populations. Sample size (N), number of haplotypes (H), haplotype diversity (Hd) and nucleotide diversity (π) in the CR region for the 400 bp long fragment, and for the 190 bp long fragment including all populations.

Geographic origin	Status	400 bp				190 bp			
		N	H	Hd	π	N	H	Hd	π
Svalbard, Norway	Wild	27	3	0.570	0.002	27	1	0.000	0.000
Kolguev, Russia	Domestic	24	6	0.728	0.009	24	6	0.728	0.013
Novaia Zemlia, Russia	Wild	20	5	0.632	0.018	20	4	0.626	0.029
Belyi Island, Russia	Wild	22	6	0.814	0.018	22	6	0.814	0.026
Pechora River, Russia	Wild	14	12	0.978	0.019	14	10	0.956	0.027
Peza River, Russia	Wild	15	6	0.800	0.018	15	6	0.800	0.030
Franz Josef Land, Russia	Wild	-	-	-	-	15	3	0.257	0.009
Total	-	122	30	0.910	0.019	137	24	0.820	0.027

**Fig 2:**
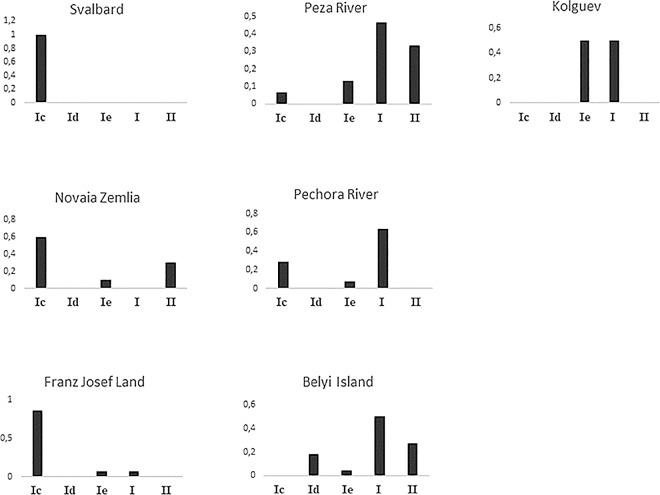
Frequencies of CR haplotype clusters in the sampled reindeer populations. Frequencies of haplotypes belonging to sub-cluster **Ic**, **Id**, **Ie** and cluster **II** in all seven populations. Haplotypes that did not cluster with any of the previously described clusters were placed in cluster **I**. Haplotype frequencies are calculated from the 400 bp long fragment for all populations, except haplotype frequencies in the ancient material from Franz Josef Land, which were calculated from the 190 bp long fragment. Haplotype frequencies show that **Ic** haplotypes are common on Svalbard, Novaia Zemlia and in the ancient material from Franz Josef Land. **Ic** haplotypes are also found in the Pechora- and Peza River populations, but are absent in the domestic reindeer population sampled on Kolguev.

We identified four previously defined haplotype clusters denoted sub-cluster **Ic**, **Id**, **Ie** and cluster **II** [26, 67, 68] (Fig 1B), all showing high support in the Bayesian phylogeny (posterior probability ≥ 99, S1 Fig), except sub-cluster **Ic** showing an intermediate level of support (posterior probability = 71). In the present study, we found sub-cluster **Ic** to comprise the 3 haplotypes previously found in Svalbard reindeer (n = 27), but also to include haplotypes found on Novaia Zemlia (n = 12), the Pechora River (n = 3), the Peza River (n = 1) and Franz Josef Land (n = 13) (Figs 1B and 2). Haplotypes in sub-cluster **Id** are commonly found in Russian domestic reindeer, but have also been identified in wild reindeer from Taimyr [26]. In the current study, we identified one haplotype belonging to sub-cluster **Id** in four wild reindeer from Belyi Island (Figs 1B and 2). **Ie** haplotypes are also commonly found in Russian domestic reindeer [26]. However, in the present study, **Ie** haplotypes were found in samples from Kolguev (n = 12), Belyi Island (n = 1), Peza River (n = 2), Pechora River (n = 1), Novaia Zemlia (n = 2) and Franz Josef Land (n = 1) (Figs 1B and 2). Finally, haplotypes in cluster **II** have previously been known to dominate in Scandinavian domestic reindeer [69]. We found cluster **II** haplotypes in wild reindeer from Peza River (n = 5), Novaia Zemlia (n = 6) and on Belyi Island (n = 6) (Figs 1B and 2). One of the three cluster **II** haplotypes found in the current study is identical to a cluster **II** haplotype commonly found in Scandinavia [26, 69].

Sub-cluster **Ic** appear to have experienced a recent population expansion as the mismatch distribution analysis showed no significant deviation from the expected distribution under the sudden expansion model (see S2 Fig). An expansion was further supported by a non-significant Harpending Raggedness index (0.071, p = 0.600) and SSD value (0.010, p = 0.710). Finally, Fu`s Fs and Ramos-Onsins R_2_ were highly significant with Fs = 5.948 (p = 0.000) and R_2_ = 0.103 (p = 0.002) adding support for a demographic expansion for this sub-cluster. Tajima`s D was negative (-1.018), but not significant (p = 0.173). Date since expansion for sub-cluster **Ic** was calculated to 5862 years before present (YBP) (95% CI: 535–10375) based on the mean number of pairwise differences, τ (τ = 2.762).

The 190 bp long fragment, including all seven populations, showed high levels of variation (Hd = 0.820, π = 0.027) and 24 haplotypes were identified. However, low levels of genetic variation was found in the ancient Franz Josef Land population (Hd = 0.257, π = 0.009) (Table 1). Three haplotypes were found here, with one individual in sub-cluster **Ie,** 13 in sub-cluster **Ic**, and one sample with a haplotype unique for Franz Josef Land (Figs 1B and 2).

## Discussion

Our results show that the most common haplotype found on Svalbard is also the most common haplotype found among the contemporary wild population on the South Islands of Novaia Zemlia and the extinct population on Franz Josef Land–suggesting that the population history of wild reindeer on these High Arctic islands was tightly linked. The genetic similarity between these archipelagic reindeer populations indicate gene flow and dispersal from a common source population, and supports the theory that all of these islands were likely colonized from the Eurasian mainland. The hypothesis of an eastern colonization of the Arctic archipelagoes is further supported by a genetic connection between the three archipelagic populations and one individual from Pechora River (Komi Republic), who shared the most common haplotype found on Svalbard, Novaia Zemlia and Franz Josef Land. Also, three similar haplotypes were observed in single samples from wild reindeer in both Pechora river basin and in the Peza river basin. However, the presence of **Ic** haplotypes in the two mainland populations could be the result of random haplotype survival from a common refugial population. More extensive sampling of Russian wild reindeer, with a special emphasis on islands in northern Siberia, would be necessary to clarify this further.

### Natural- or human induced dispersal

Published radiocarbon dates of archaic bones collected on Franz Josef Land suggest that wild reindeer populated the archipelago 6400–1300 YBP [36]. These results fit with the dates we obtained for our own radiocarbon dated antler samples from the same islands (>2000 years). Pollen studies of archaic reindeer pellets found in peat cores on Svalbard indicate that reindeer colonized this archipelago between 6700 and 5000 YBP [70, 71]. The history of colonization of Novaia Zemlia by reindeer for much of Holocene remains to be documented. However, geomorphologic studies suggest that by the time wild reindeer were present on Svalbard and Franz Josef Land, Novaia Zemlia was also de-glaciated and therefore open for natural colonization as well [72, 73]. The early colonization of Svalbard and Franz Josef Land, and the strong genetic link found between the ancient Franz Josef Land samples and the contemporary populations on Svalbard and Novaia Zemlia, both imply that the Arctic reindeer type lived on all of these archipelagos long before humans approached the region [74].

We did find one individual on Novaia Zemlia with a haplotype also found in reindeer on Kolguev Island (sub-cluster **Ie**). This finding can be explained by the recent translocation of a small number of domestic reindeer from Kolguev to Novaia Zemlia, between 1928 and 1931 [41]. The weak genetic connection found between the wild populations on Novaia Zemlia and the present-day domestic population on Kolguev Island, together with the genetic similarities found between Svalbard, Novaia Zemlia and Franz Josef Land, imply that the maternal genetic structure of northern archipelagic reindeer populations, including present populations on Novaia Zemlia and Svalbard, is mainly indigenous.

### Post glacial colonization of Eurasian arctic archipelagos

Our results indicate an eastern colonization route of the Eurasian arctic archipelagos. The Bering land refuge has traditionally been most widely discussed as a single origin point for various continental distributions of *Rangifer* [19, 75] as well as several other circumpolar species [76]. However, recently there has been a discussion of the importance of the role of a lesser-known set of refugia in the High Arctic of Western Siberia. Fedorov et al. [77] performed a circumpolar phylogeographic analysis of lemmings (*Lemmus*) questioning the centrality of the traditional Beringian refuge for the post-glacial re-colonization of the Arctic. They demonstrate how four different mtDNA linages of the circumpolar lemming (*Lemmus*) indicate separation by glacial barriers, followed by post glacial colonization from refugia other than Beringia. Shaefer et al. [78] in their recent review of North American mtDNA phylogeographic analyses for multiple circumpolar species point to additional complexity of multiple “refugia within refugia” within and between ice sheets. Salonen, Seppä [79] in their review of the palynological literature for Western Siberia point to possible refuge located in the Pechora River basin. The existence of alternative refugia, separated from Beringia, might be one explanation for the unique genetic composition observed in Eurasian archipelagic reindeer. On the other hand, our results suggest that there was a population expansion of sub-cluster **Ic** as recently as 5000–6000 YBP. This implies that the unique genetic composition of these Arctic reindeer populations may have resulted from bottlenecking, isolation, and then subsequent expansion in the High Arctic well after the retreat of the ice, rather than isolation in alternative refugia during the last glacial maximum (LGM). Further studies of the genetic structure in Russian reindeer populations would help to answer this problem.

Post-glacial range shifts and the expansion of reindeer populations, as well as those of other cold-adapted species, is probably connected to the major environmental changes taking place in northern Eurasia during the Holocene [80]. Sea surface conditions in the southeastern Barents Sea region reconstructed from dinoflagellate cyst assemblages, indicate a warm and stable climate between 8000–5000 YBP [81]. These correlate well with other terrestrial and marine records of climate conditions during this period [81], as well as estimates indicating that the spruce (*Picea*) and birch (*Betula*) tree lines in northern Eurasia were located at least 150 km further North from their present location, and in the case of birch, may have reached the seacoast [36, 79]. The expanding forest may have driven Arctic-adapted reindeer populations to migrate further north to seek open tundra landscapes. The warmer climate would have facilitated growth of various vascular plants in the high arctic, thus expanding the food base for reindeer on the Eurasian arctic archipelagos [71]. As mentioned above, there are anecdotal accounts of the movement of large-bodied reindeer from the Eurasian mainland to Svalbard in historic times [34], suggesting that such migrations between islands over the ice are possible. This scenario is supported by the shared haplotypes held by wild reindeer in Taimyr and in Svalbard by Gravlund and colleagues [9], and our own discovery of shared haplotypes between the three arctic archipelagos and the mainland populations from the Peza and Pechora River basins in the current study.

## Concluding Remarks

*Rangifer* are in many ways a classic circumpolar species providing an important anchor to the environmental history of the High Arctic, and also to the lives of local people. Contemporary climate change would be expected to alter the distribution and demography of *Rangifer* today as has been the case in the past. Mammals living in the High Arctic have limited opportunities to migrate further north. To survive, they will have to depend on their ability to adapt where they are. Therefore they are under particular risk. This necessitates having proper management plans with an emphasis on conserving genetic variability for indigenous archipelagic reindeer.

This study has established certain strong genetic similarities found between wild reindeer populations on Svalbard, Novaia Zemlia and Franz Josef Land implying that the maternal genetic structure of these archipelagic reindeer populations is indigenous and unique. The study lends considerable weight to the hypothesis that these islands may have colonized from the Eurasian mainland via an eastern route. Moreover the study strongly disproves the suggestion that populations for example on Novaia Zemlia are feral populations of introduced domestic reindeer. It is our hope that these important results will help clarify existing conservation plans for wild reindeer on Novaia Zemlia in the Russian Arctic Strict Nature Reserve and its aim at conserving this important and unique population.

## Supporting Information

S1 Fig Bayesian phylogeny based on the 400 bp long fragment, excluding the ancient samples from Franz Josef Land. The Bayesian phylogeny shows 30 control region haplotypes and support for sub-cluster **Ic**, **Id**, **Ie** and **II** (posterior probability values ≥70 is shown at each node).
(TIF)

S2 Fig Mismatch distribution. The observed pairwise difference (blue bars) and the expected mismatch distribution (red bars) under the sudden expansion model among individuals in sub-cluster **Ic**. The mismatch analyses show a unimodal distribution, which is characteristic for a recently expanded population [63].
(TIF)

S1 Table ^14^C and calibrated radiocarbon dates on antler samples from Franz Josef Land. The ^14^C dates were calibrated using CALIB 6.1.1 [53], based on the data set IntCal13 [54] with 2σ ranges.
(PDF)

S2 Table Sample information and NCBI GenBank accession numbers. (PDF)

S1 Text Boiling method for DNA extraction. (PDF)
