# Supplementary figures and images for: Colonizing the High Arctic: Mitochondrial DNA Reveals Common Origin of Eurasian Archipelagic Reindeer (Rangifer tarandus)
Source: PLoS One. 2016 Nov 23;11(11):e0165237. doi: 10.1371/journal.pone.0165237 (PMC5120779; doi:10.1371/journal.pone.0165237)

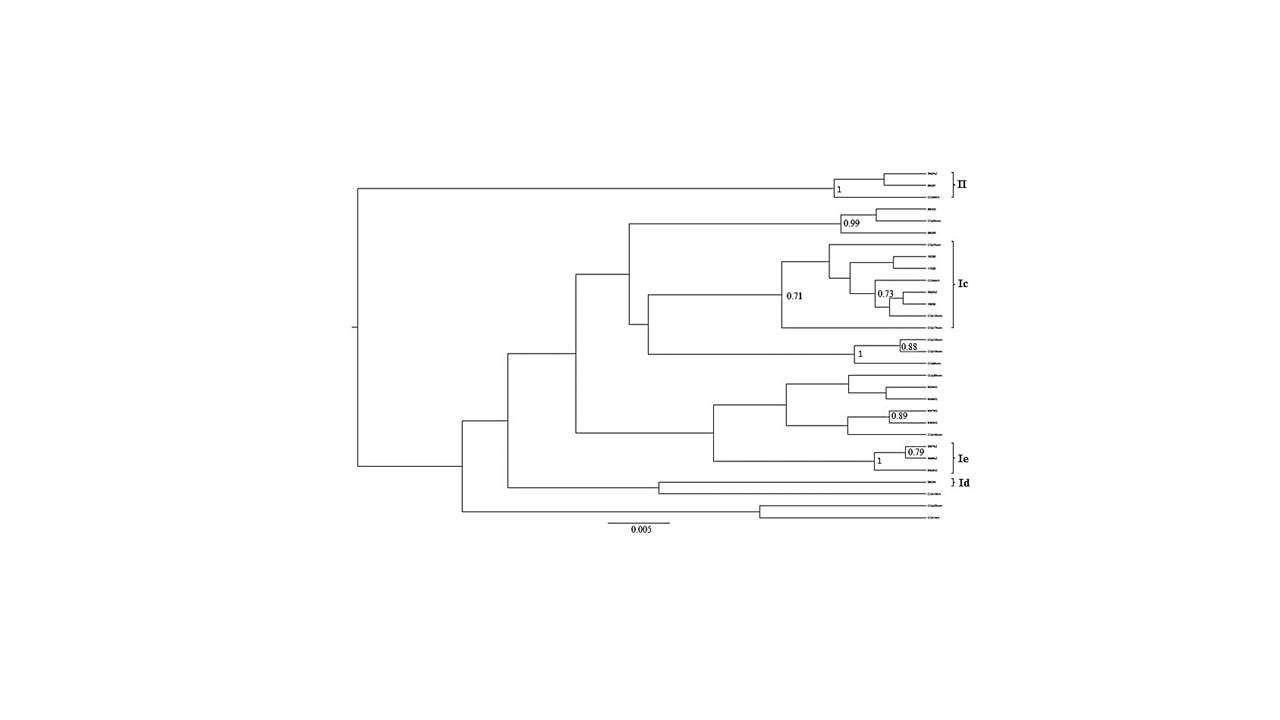

Supplement: S1 Fig — The Bayesian phylogeny shows 30 control region haplotypes and support for sub-cluster Ic, Id, Ie and II (posterior probability values ≥70 is shown at each node). (TIF) [file pone.0165237.s001.tif]

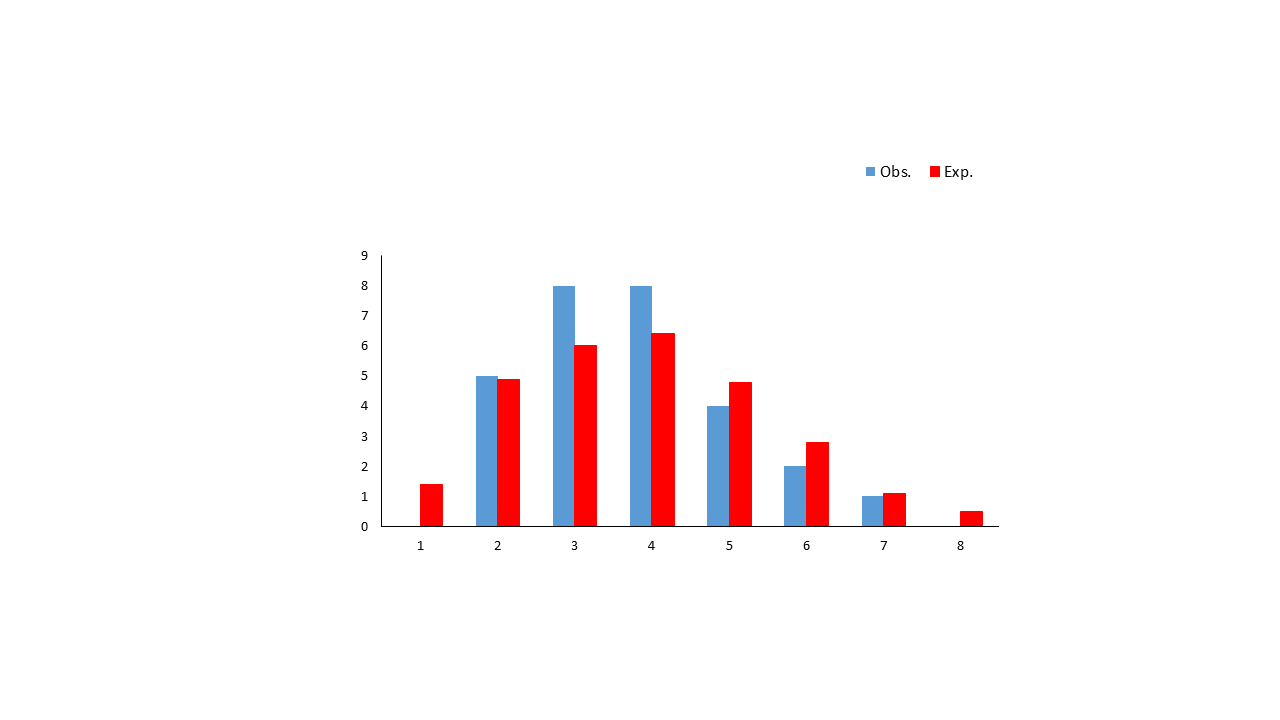

Supplement: S2 Fig — The observed pairwise difference (blue bars) and the expected mismatch distribution (red bars) under the sudden expansion model among individuals in sub-cluster Ic. The mismatch analyses show a unimodal distribution, which is characteristic for a recently expanded population [63]. (TIF) [file pone.0165237.s002.tif]
