# Supplementary material for: Colonizing the High Arctic: Mitochondrial DNA Reveals Common Origin of Eurasian Archipelagic Reindeer (Rangifer tarandus)
Source: PLoS One. 2016 Nov 23;11(11):e0165237. doi: 10.1371/journal.pone.0165237 (PMC5120779; doi:10.1371/journal.pone.0165237)
Supplement: S1 Table — The 14C dates were calibrated using CALIB 6.1.1 [53], based on the data set IntCal13 [54] with 2σ ranges. (PDF) [file pone.0165237.s003.pdf]

| Sample ID | Site | Calibrated date | Lab code |
|---|---|---|---|
| T-2580 | Franz Josef Land | BP 2 468 ± 31 | Ua-49137 |
| T-2594 | Franz Josef Land | BP 3 806 ± 32 | Ua-49138 |
| T2598 | Franz Josef Land | BP 2 390 ± 30 | Ua4-9139 |
| T-2600 | Franz Josef Land | BP  3 835 ± 32 | Ua-49140 |
