# Supplementary material for: Colonizing the High Arctic: Mitochondrial DNA Reveals Common Origin of Eurasian Archipelagic Reindeer (Rangifer tarandus)
Source: PLoS One. 2016 Nov 23;11(11):e0165237. doi: 10.1371/journal.pone.0165237 (PMC5120779; doi:10.1371/journal.pone.0165237)
Supplement: S2 Table — (PDF) [file pone.0165237.s004.pdf]

**Sampling location**	**Federal subject**	**Country**	**Status**	**Id. nb.**	**Accession nb.**
Nordenskiöld Land, Svalbard		Norway	Wild	Re115	KX094711
Nordenskiöld Land, Svalbard		Norway	Wild	Re116	KX094712
Nordenskiöld Land, Svalbard		Norway	Wild	Re117	KX094713
Nordenskiöld Land, Svalbard		Norway	Wild	Re118	KX094714
Nordenskiöld Land, Svalbard		Norway	Wild	Re119	KX094715
Nordaustlandet Svalbard		Norway	Wild	Re189	KX094716
Nordaustlandet Svalbard		Norway	Wild	Re190	KX094717
Nordaustlandet Svalbard		Norway	Wild	Re191	KX094718
Nordaustlandet Svalbard		Norway	Wild	Re192	KX844748
Nordaustlandet Svalbard		Norway	Wild	Re193	KX094719
Nordaustlandet Svalbard		Norway	Wild	Re194	KX844749
Nordaustlandet Svalbard		Norway	Wild	Re195	KX094720
Nordaustlandet Svalbard		Norway	Wild	Re196	KX094721
Nordaustlandet Svalbard		Norway	Wild	Re197	KX094722
Nordenskiöld Land, Svalbard		Norway	Wild	Re344	KX094723

Nordenskiöld Land, Svalbard		Norway	Wild	Re346	KX094724
Nordenskiöld Land, Svalbard		Norway	Wild	Re5189	KX094698
Nordenskiöld Land, Svalbard		Norway	Wild	Re5192	KX094701
Nordenskiöld Land, Svalbard		Norway	Wild	Re5193	KX094702
Nordenskiöld Land, Svalbard		Norway	Wild	Re5194	KX094703
Nordenskiöld Land, Svalbard		Norway	Wild	Re5195	KX094704
Nordenskiöld Land, Svalbard		Norway	Wild	Re5196	KX094705
Nordenskiöld Land, Svalbard		Norway	Wild	Re5198	KX094707
Nordenskiöld Land, Svalbard		Norway	Wild	Re5199	KX094708
Nordenskiöld Land, Svalbard		Norway	Wild	Re5202	KX094709
Nordenskiöld Land, Svalbard		Norway	Wild	Re5203	KX094710
Nordenskiöld Land, Svalbard		Norway	Wild	Re5205	KX844750
Novaia Zemlia, South Island	Arkhangelsk oblast	Russia	Wild	Re5977	KX844728
Novaia Zemlia, South Island	Arkhangelsk oblast	Russia	Wild	Re5979	KX844729
Novaia Zemlia, South Island	Arkhangelsk oblast	Russia	Wild	Re5980	KX844730
Novaia Zemlia, South Island	Arkhangelsk oblast	Russia	Wild	Re5982	KX844731

Novaia Zemlia, South Island	Arkhangelsk oblast	Russia	Wild	Re5984	KX844732
Novaia Zemlia, South Island	Arkhangelsk oblast	Russia	Wild	Re5985	KX844733
Novaia Zemlia, South Island	Arkhangelsk oblast	Russia	Wild	Re5986	KX844734
Novaia Zemlia, South Island	Arkhangelsk oblast	Russia	Wild	Re5987	KX844735
Novaia Zemlia, South Island	Arkhangelsk oblast	Russia	Wild	Re5988	KX844736
Novaia Zemlia, South Island	Arkhangelsk oblast	Russia	Wild	Re5990	KX844737
Novaia Zemlia, South Island	Arkhangelsk oblast	Russia	Wild	Re5992	KX844738
Novaia Zemlia, South Island	Arkhangelsk oblast	Russia	Wild	Re5994	KX844739
Novaia Zemlia, South Island	Arkhangelsk oblast	Russia	Wild	Re5995	KX844740
Novaia Zemlia, South Island	Arkhangelsk oblast	Russia	Wild	Re5996	KX844741
Novaia Zemlia, South Island	Arkhangelsk oblast	Russia	Wild	Re5997	KX844742
Novaia Zemlia, South Island	Arkhangelsk oblast	Russia	Wild	Re5998	KX844743
Novaia Zemlia, South Island	Arkhangelsk oblast	Russia	Wild	Re5999	KX844744
Novaia Zemlia, South Island	Arkhangelsk oblast	Russia	Wild	Re6000	KX844745
Novaia Zemlia, South Island	Arkhangelsk oblast	Russia	Wild	Re6002	KX844746
Novaia Zemlia, South Island	Arkhangelsk oblast	Russia	Wild	Re6003	KX844747

Kolguev Island	Nenets Autonomous Okrug	Russia	Domestic	Re6044	KX844751
Kolguev Island	Nenets Autonomous Okrug	Russia	Domestic	Re6045	KX844752
Kolguev Island	Nenets Autonomous Okrug	Russia	Domestic	Re6046	KX844753
Kolguev Island	Nenets Autonomous Okrug	Russia	Domestic	Re6047	KX844754
Kolguev Island	Nenets Autonomous Okrug	Russia	Domestic	Re6048	KX844755
Kolguev Island	Nenets Autonomous Okrug	Russia	Domestic	Re6049	KX844756
Kolguev Island	Nenets Autonomous Okrug	Russia	Domestic	Re6050	KX844757
Kolguev Island	Nenets Autonomous Okrug	Russia	Domestic	Re6051	KX844758
Kolguev Island	Nenets Autonomous Okrug	Russia	Domestic	Re6052	KX844759
Kolguev Island	Nenets Autonomous Okrug	Russia	Domestic	Re6053	KX844760
Kolguev Island	Nenets Autonomous Okrug	Russia	Domestic	Re6054	KX844761
Kolguev Island	Nenets Autonomous Okrug	Russia	Domestic	Re6055	KX844762
Kolguev Island	Nenets Autonomous Okrug	Russia	Domestic	Re6056	KX844763
Kolguev Island	Nenets Autonomous Okrug	Russia	Domestic	Re6057	KX844764
Kolguev Island	Nenets Autonomous Okrug	Russia	Domestic	Re6058	KX844765
Kolguev Island	Nenets Autonomous Okrug	Russia	Domestic	Re6059	KX844766

Kolguev Island	Nenets Autonomous Okrug	Russia	Domestic	Re6060	KX844767
Kolguev Island	Nenets Autonomous Okrug	Russia	Domestic	Re6061	KX844768
Kolguev Island	Nenets Autonomous Okrug	Russia	Domestic	Re6062	KX844769
Kolguev Island	Nenets Autonomous Okrug	Russia	Domestic	Re6063	KX844770
Kolguev Island	Nenets Autonomous Okrug	Russia	Domestic	Re6065	KX844771
Kolguev Island	Nenets Autonomous Okrug	Russia	Domestic	Re6066	KX844772
Kolguev Island	Nenets Autonomous Okrug	Russia	Domestic	Re6067	KX844773
Kolguev Island	Nenets Autonomous Okrug	Russia	Domestic	Re6068	KX844774
Pechora River	Komi Republic	Russia	Wild	COp5-Komi	JQ073832
Pechora River	Komi Republic	Russia	Wild	COp7-Komi	JQ073843
Pechora River	Komi Republic	Russia	Wild	COp8-Komi	JQ073844
Pechora River	Komi Republic	Russia	Wild	COp10-Komi	JQ073840
Pechora River	Komi Republic	Russia	Wild	COp11-Komi	JQ073833
Pechora River	Komi Republic	Russia	Wild	COp12-Komi	JQ073842
Pechora River	Komi Republic	Russia	Wild	COp13-Komi	JQ073841
Pechora River	Komi Republic	Russia	Wild	COp16-Komi	JQ073845

Pechora River	Komi Republic	Russia	Wild	COp17-Komi	JQ073847
Pechora River	Komi Republic	Russia	Wild	COp22-Komi	JQ073846
Pechora River	Komi Republic	Russia	Wild	COp23-Komi	JQ073832
Pechora River	Komi Republic	Russia	Wild	COp24-Komi	JQ073838
Pechora River	Komi Republic	Russia	Wild	COp25-Komi	JQ073838
Pechora River	Komi Republic	Russia	Wild	COp26-Komi	JQ073839
Mezen River	Arkhangelsk oblast	Russia	Wild	COA1-Arh	JQ073835
Mezen River	Arkhangelsk oblast	Russia	Wild	COA4-Arh	JQ073836
Mezen River	Arkhangelsk oblast	Russia	Wild	COA6-Arh	JQ073833
Mezen River	Arkhangelsk oblast	Russia	Wild	COA7-Arh	JQ073832
Mezen River	Arkhangelsk oblast	Russia	Wild	COA8-Arh	JQ073834
Mezen River	Arkhangelsk oblast	Russia	Wild	COA9-Arh	JQ073834
Mezen River	Arkhangelsk oblast	Russia	Wild	COA10-Arh	JQ073832
Mezen River	Arkhangelsk oblast	Russia	Wild	11a-Arh	JQ073832
Mezen River	Arkhangelsk oblast	Russia	Wild	12a-Arh	JQ073832
Mezen River	Arkhangelsk oblast	Russia	Wild	15a-Arh	JQ073833

Mezen River	Arkhangelsk oblast	Russia	Wild	16a-Arh	JQ073832
Mezen River	Arkhangelsk oblast	Russia	Wild	17a-Arh	JQ073834
Mezen River	Arkhangelsk oblast	Russia	Wild	18a-Arh	JQ073834
Mezen River	Arkhangelsk oblast	Russia	Wild	COA19-Arh	JQ073837
Mezen River	Arkhangelsk oblast	Russia	Wild	22a-Arh	JQ073834
Belyi Island, Yamal Nenets	Tiumen Oblast	Russia	Wild	Re6281	KX844796
Belyi Island, Yamal Nenets	Tiumen Oblast	Russia	Wild	Re6282	KX844795
Belyi Island, Yamal Nenets	Tiumen Oblast	Russia	Wild	Re6283	KX844794
Belyi Island, Yamal Nenets	Tiumen Oblast	Russia	Wild	Re6284	KX844793
Belyi Island, Yamal Nenets	Tiumen Oblast	Russia	Wild	Re6285	KX844792
Belyi Island, Yamal Nenets	Tiumen Oblast	Russia	Wild	Re6286	KX844791
Belyi Island, Yamal Nenets	Tiumen Oblast	Russia	Wild	Re6287	KX844790
Belyi Island, Yamal Nenets	Tiumen Oblast	Russia	Wild	Re6288	KX844789
Belyi Island, Yamal Nenets	Tiumen Oblast	Russia	Wild	Re6289	KX844788
Belyi Island, Yamal Nenets	Tiumen Oblast	Russia	Wild	Re6290	KX844787
Belyi Island, Yamal Nenets	Tiumen Oblast	Russia	Wild	Re6291	KX844786

Belyi Island, Yamal Nenets	Tiumen Oblast	Russia	Wild	Re6293	KX844785
Belyi Island, Yamal Nenets	Tiumen Oblast	Russia	Wild	Re6294	KX844784
Belyi Island, Yamal Nenets	Tiumen Oblast	Russia	Wild	Re6295	KX844783
Belyi Island, Yamal Nenets	Tiumen Oblast	Russia	Wild	Re6296	KX844782
Belyi Island, Yamal Nenets	Tiumen Oblast	Russia	Wild	Re6297	KX844781
Belyi Island, Yamal Nenets	Tiumen Oblast	Russia	Wild	Re6299	KX844780
Belyi Island, Yamal Nenets	Tiumen Oblast	Russia	Wild	Re6300	KX844779
Belyi Island, Yamal Nenets	Tiumen Oblast	Russia	Wild	Re6301	KX844778
Belyi Island, Yamal Nenets	Tiumen Oblast	Russia	Wild	Re6302	KX844777
Belyi Island, Yamal Nenets	Tiumen Oblast	Russia	Wild	Re6303	KX844776
Belyi Island, Yamal Nenets	Tiumen Oblast	Russia	Wild	Re6304	KX844775
Hooker/Hays islands, Franz Josef Land	Arkhangelsk oblast	Russia	Wild	F2580	KX844797
Hooker/Hays islands, Franz Josef Land	Arkhangelsk oblast	Russia	Wild	F2587	KX844798
Hooker/Hays islands, Franz Josef Land	Arkhangelsk oblast	Russia	Wild	F2594	KX844799
Hooker/Hays islands, Franz Josef Land	Arkhangelsk oblast	Russia	Wild	F2598	KX844800
Hooker/Hays islands, Franz Josef Land	Arkhangelsk oblast	Russia	Wild	F2600	KX844801

Hooker/Hays islands, Franz Josef Land	Arkhangelsk oblast	Russia	Wild	F2601	KX844802
Hooker/Hays islands, Franz Josef Land	Arkhangelsk oblast	Russia	Wild	F2602	KX844803
Hooker/Hays islands, Franz Josef Land	Arkhangelsk oblast	Russia	Wild	F2603	KX844804
Hooker/Hays islands, Franz Josef Land	Arkhangelsk oblast	Russia	Wild	F2604	KX844805
Hooker/Hays islands, Franz Josef Land	Arkhangelsk oblast	Russia	Wild	F2605	KX844806
Hooker/Hays islands, Franz Josef Land	Arkhangelsk oblast	Russia	Wild	F2609	KX844807
Hooker/Hays islands, Franz Josef Land	Arkhangelsk oblast	Russia	Wild	F2676	KX844808
Hooker/Hays islands, Franz Josef Land	Arkhangelsk oblast	Russia	Wild	F2677	KX844809
Hooker/Hays islands, Franz Josef Land	Arkhangelsk oblast	Russia	Wild	F2678	KX844810
Hooker/Hays islands, Franz Josef Land	Arkhangelsk oblast	Russia	Wild	F2679	KX844811
