# Supplementary material for: Colonizing the High Arctic: Mitochondrial DNA Reveals Common Origin of Eurasian Archipelagic Reindeer (Rangifer tarandus)
Source: PLoS One. 2016 Nov 23;11(11):e0165237. doi: 10.1371/journal.pone.0165237 (PMC5120779; doi:10.1371/journal.pone.0165237)
Supplement: S1 Text — (PDF) [file pone.0165237.s005.pdf]

100 µl EDTA blood was added 1000 µl lysis buffer. The samples were vortexed before they were centrifuged at 13000 revolutions per minute (rpm) for 1 minute and the supernatant was removed. This step was repeated once before washing the pellet with 1000 µl Tris-EDTA (TE) buffer (pH 8) and centrifuged at 13000 rpm for 1 minute. The supernatant was removed and the pellet dissolved with 100µl PCR buffer with detergent containing 1 µl proteinase K (10 mg/ml) pr. 100 µl buffer. The samples were incubated on a thermomixer for 2 hours at 56 degrees and 700 rpm before inactivation of proteinase K by incubation for 10 minutes at 95 degrees.
